# Creating electronic oscillator-based Ising machines without external injection locking

**DOI:** 10.1038/s41598-021-04057-2

**Published:** 2022-01-19

**Authors:** Jaykumar Vaidya, R. S. Surya Kanthi, Nikhil Shukla

**Affiliations:** Department of Electrical and Computer Engineering, University of Virginia, Charlottesville, VA 22904 USA

**Keywords:** Electrical and electronic engineering, Computer science

## Abstract

Coupled electronic oscillators have recently been explored as a compact, integrated circuit- and room temperature operation-compatible hardware platform to design Ising machines. However, such implementations presently require the injection of an externally generated second-harmonic signal to impose the phase bipartition among the oscillators. In this work, we experimentally demonstrate a new electronic autaptic oscillator (EAO) that uses engineered feedback to eliminate the need for the generation and injection of the external second harmonic signal to minimize the Ising Hamiltonian. Unlike conventional relaxation oscillators that typically decay with a single time constant, the feedback in the EAO is engineered to generate two decay time constants which effectively helps generate the second harmonic signal internally. Using this oscillator design, we show experimentally, that a system of capacitively coupled EAOs exhibits the desired bipartition in the oscillator phases without the need for any external second harmonic injection, and subsequently, demonstrate its application in solving the computationally hard Maximum Cut (MaxCut) problem. Our work not only establishes a new oscillator design aligned to the needs of the oscillator Ising machine but also advances the efforts to creating application specific analog computing platforms.

## Introduction

The Ising model, originally developed for spin glass systems^1^, has recently experienced renewed attention owing to its application in accelerating computationally hard problems which are still considered intractable to solve using conventional digital computers. This is motivated by the fact that a large number of such problems^2–6^ can be directly mapped to the Ising Hamiltonian: ${\text{H}} = -\sum_{i,j}^{N}{J}_{ij}{\sigma }_{i}{\sigma }_{j}$, where ${\sigma }_{i}$ is the *i*th spin ($\pm 1$), ${J}_{ij}$ is interaction coefficient between spin *i* and spin *j*, and *N* is the total number of spins in the system; the self-interaction term ($-\sum_{i}^{N}{h}_{i}{\sigma }_{i}$) in the Ising Hamiltonian has been neglected here. For instance, consider the combinatorial optimization-based Maximum Cut (MaxCut) problem—the benchmark problem considered in this work—which entails computing a cut that divides the nodes of the graph in two sets (S1, S2) such that the number of common edges among the two sets is as large as possible. This problem can be mapped to $H$ by considering each node of the graph as a spin. When a node belongs to S1 (S2), then it is assigned a value (spin) + 1 (− 1), respectively; further, considering only binary weights (relevant to unweighted graphs, considered here), ${J}_{ij}=-1$, if an edge exists between nodes *i* and *j*; else ${J}_{ij}=0$. This ensures that if the two adjacent nodes (i.e., nodes connected by an edge) are in the same set, then ${J}_{ij}{\sigma }_{i}{\sigma }_{j}=-1$; if they are in different sets, then ${J}_{ij}{\sigma }_{i}{\sigma }_{j}=1$. Thus, computing the MaxCut of the graph entails finding a configuration of nodes (spins) that minimizes $-\sum_{i,j}^{N}{J}_{ij}{\sigma }_{i}{\sigma }_{j}$, which is equivalent to minimizing the Ising Hamiltonian ($H$). Consequently, this has motivated an active effort to realize a physical Ising machine that evolves to minimize its energy (proportional to $H$) and attain the ground state, which in turn, should correspond to the optimal solution of the MaxCut problem. Examples of approaches that have been explored to implement Ising machines include quantum annealing (D-Wave)^7–9^, quantum mechanical Kerr parametric oscillators^10,11^, optical parametric oscillator based Coherent Ising machines (CIMs)^12–15^, electromechanical CIMs^16^, processors based on annealing in-/near-memory^17–19^, SRAM based Ising machines that rely on CMOS annealing^20^, memristor-based implementations^21^, and coupled electronic oscillators- the focus of the present work. Coupled electronic oscillators have recently been investigated as a promising approach for developing Ising machines since oscillators, in principle, can be made low-power, compact^22–32^, compatible with room-temperature operation^33,34^, and can be manufactured using integrated circuit technology^2,3,35^.

To solve the MaxCut problem directly using the oscillator hardware, each node of the graph is mapped to an oscillator and every edge is represented by a coupling capacitor. The resulting phases exhibit a bipartition (0° or 180°) that can be mapped to the sets S1 and S2 created by the (Max-)Cut. However, coercing the oscillator phases to exhibit the bipartion requires the injection of a second harmonic signal i.e., f_inj_ $\cong$ 2 × f_R_ (f_inj_ = frequency of injected signal; f_R_ = frequency of resonant circuit) to every oscillator in the network^4^. In this work, we propose a novel electronic autaptic oscillator (EAO) design that eliminates the need for second harmonic injection; the prefix ‘autaptic’ is inspired from the autapse structures found in biological spiking neurons which are synapses from the excitatory neuron onto itself (unlike their usual synaptic counterparts that connect to a different neuron) and provide feedback to regulate the neuron’s spiking activity^36–39^. The autaptic feature implemented in the EAO through electronic feedback, introduces two decay time constants during the relaxation phase. These dual time constants can be engineered to effectively create the second harmonic signal required to impose the phase bipartition, thus, eliminating the need for an external second harmonic signal source.

## Results

**Figure 1:**
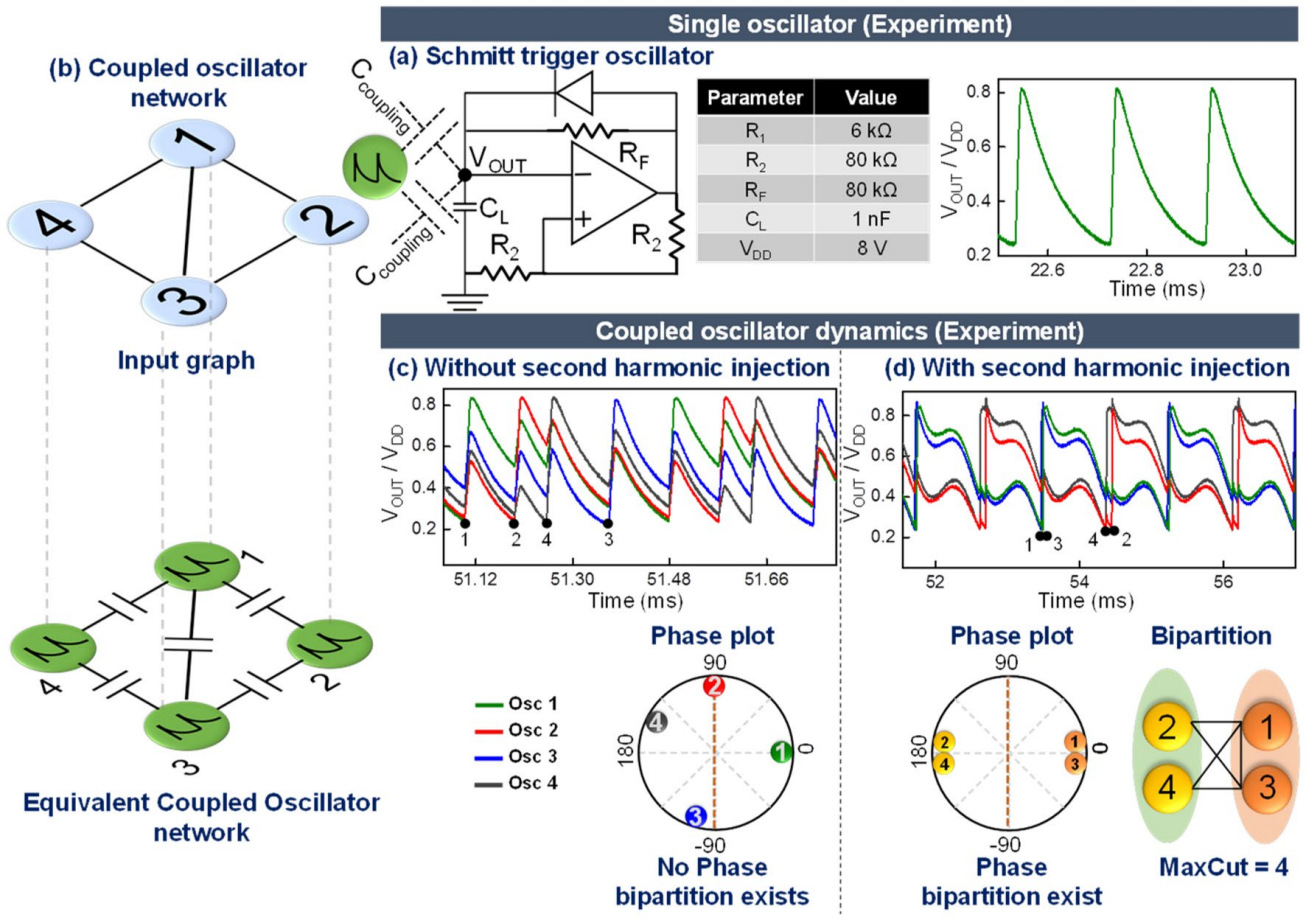
Coupled oscillators as an Ising machine. (**a**) Schematic of the Schmitt-trigger based relaxation oscillator and the corresponding experimentally measured time domain output. (**b**) Illustrative graph, and the corresponding coupled oscillator circuit implementation to compute the MaxCut. (**c**,**d**) Time domain outputs and corresponding phase plots for a coupled system of oscillators (corresponding to the input graph on the left) with and without external second harmonic injection, respectively. It can be observed that the second harmonic signal is needed to realize the (Ising model-relevant) bipartition in the oscillator phases, required to solve the MaxCut problem. Without the second harmonic signal, the oscillator phases exhibit a continuous phase ordering.

The role of second harmonic injection is illustrated using the representative 4 node graph shown in Fig. 1. Figure 1a shows the schematic and experimentally measured time domain output of the Schmitt-trigger based relaxation oscillator used in the experiments. The diode in the feedback circuit is used to create an asymmetric output waveform with a rise time that is significantly smaller than the fall time. Subsequently, when the oscillators are coupled capacitively (C_c_ = 1nF) in a manner topologically equivalent to the input graph (Fig. 1b), it can be observed that the oscillators are frequency synchronized. Moreover, when no external signal is applied (Fig. 1c), the oscillator phases exhibit a continuous distribution as shown in the corresponding phase plot (the phase ordering, in fact, represents the independent sets of the graph^2,33,34^). However, when an external second harmonic signal (f_inj_ = 3.4 kHz) is injected (Fig. 1d), the oscillator phases exhibit a bipartition that can subsequently be shown to represent the two sets created by the MaxCut (= 4, for the graph considered in the experiment). The second harmonic signal effectively modifies the energy landscape by lowering the energy corresponding to the 0° and 180° oscillator phases^4^.

**Figure 2:**
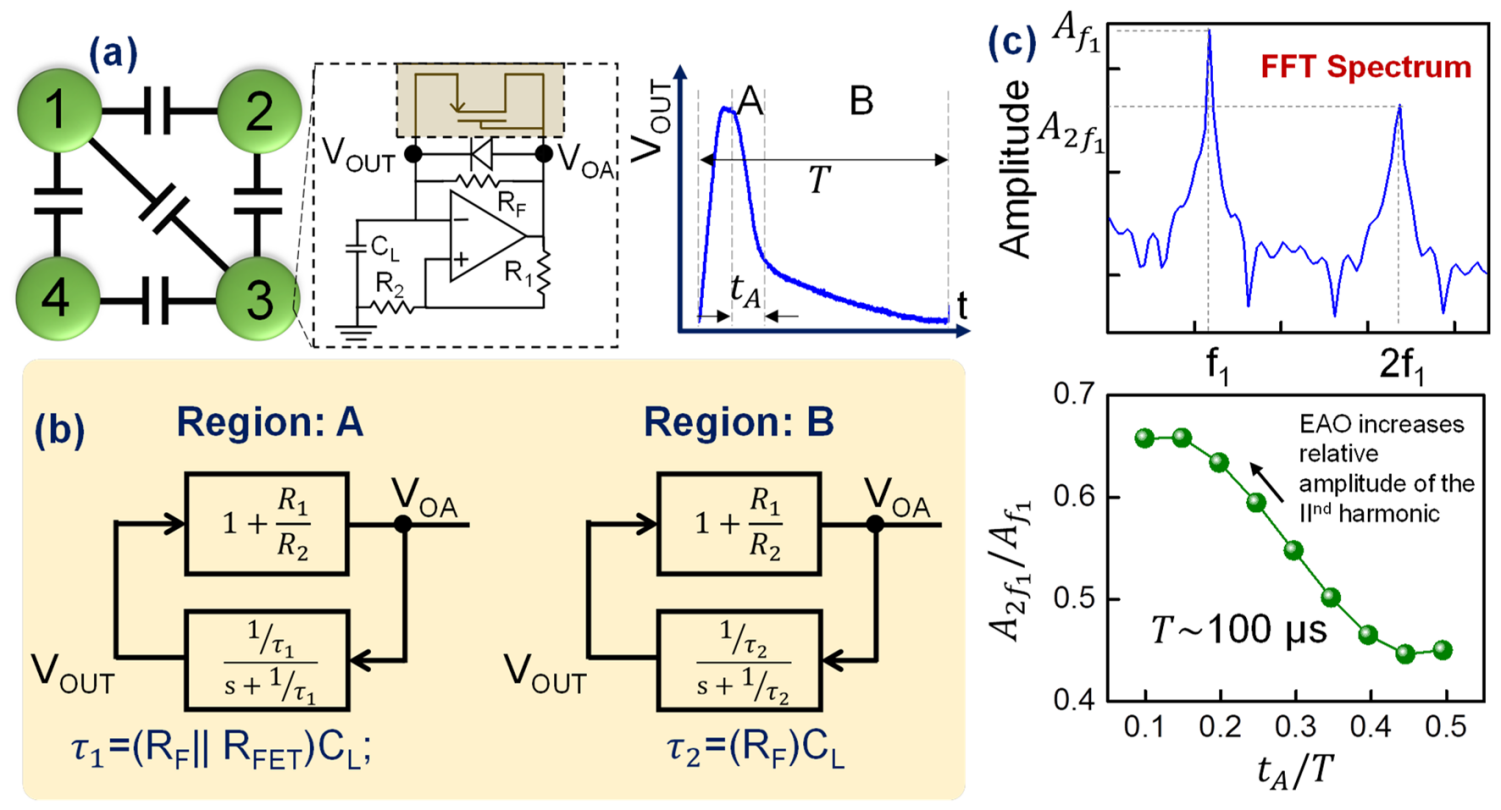
Operating principle of the EAO. (**a**) Schematic of a coupled EAO circuit with the inset showing an EAO. An illustration of the time domain output of the EAO showing the two distinct relaxation regimes (arising from different time constants). (**b**) Control system block diagram of the EAO during the two different phases of voltage relaxation (**c**) Amplitude of the second harmonic relative to that of the fundamental frequency (${A}_{{2f}_{1}}/{A}_{{f}_{1}}$) as a function of the relative time periods of the two phases observed during voltage relaxation of the EAO.

However, generating, and injecting the second harmonic injection signal to every oscillator can incur a significant energy and area overhead. We therefore explore the possibility of eliminating this requirement by redesigning the fundamental compute block of the system-the oscillator. Figure 2a shows the proposed EAO design which essentially consists of a Schmitt-trigger oscillator (similar to that proposed in Fig. 1a) with an additional non-linear feedback element—a diode connected (p-type) MOSFET. Figure 2a shows the experimentally measured time domain waveform of the oscillator where, unlike the conventional oscillator, the discharging phase reveals two distinct two-time constants. While the motivation behind modifying the oscillator dynamics will be discussed in the following sections, we first describe the origin of the two-time constants. The time constant of the oscillator is effectively determined by the net resistance and capacitance in the feedback path. Initially, during the discharging phase, the diode connected p-MOSFET is designed to be in the ON state, and hence, forms a parallel conducting path to the feedback resistance, R_F_; the diode is reverse biased and has negligible contribution to the conduction. Thus, during this initial phase, the load capacitor, C_L_, effectively discharges with a time constant τ_1_ = (R_FET_||R_F_)C_L_. As the output voltage decreases, the diode-connected MOSFET turns OFF, and the output now begins to decay with a larger time constant τ_2_ = R_F_C_L_ (τ_2_ > τ_1_) until it reaches the minimum. Subsequently, the oscillator output begins to rise again with the rise time being governed by the dynamic resistance of the diode.

To analyze the dynamics of the EAO, we consider the control block diagram of the EAO (Fig. 2). We specifically focus on voltage relaxation phase in the time domain waveform characterized by two different relaxation time constants. During this phase, the EAO can be modelled as a control system with a feed-forward gain, $G=1+\frac{{R}_{1}}{{R}_{2}}$ and feedback factor ${{\upbeta}} = \frac{{1/{{\uptau}}}}{{{\text{s}} + 1/{{\uptau}}}}$ where $\uptau =RC$. In region A, where the p-MOSFET is considered to be in the ON state, τ_1_ = (R_F_||R_FET_)C_L_, and in region B, where the p-MOSFET is OFF, τ_2_ = R_F_C_L_ (i.e., FET is considered as an open circuit). We note that while the p-MOSFET switching has been considered abrupt between the ON (with ON resistance R_FET_) and the OFF state in the above discussion for simplicity, the actual resistance evolution will be a continuum. Using this simplistic model, we analyze the frequency spectrum (Fig. 2c) of the output during the voltage relaxation, specifically focusing on the amplitude of the second harmonic relative to the fundamental frequency, i.e., ${A}_{{2f}_{1}}$/${A}_{{f}_{1}}$ (indicative of the relative power that resides at the two frequencies) as a function of the relative time period of the two relaxation phases (expressed as t_A_/T; t_A_ is the time period of the first phase of the voltage relaxation, and T is the total time period of the EAO); the relative time periods are controlled by the RC time constants ${\tau }_{1}$ and ${\tau }_{2}$. It can be observed from Fig. 2c that ${A}_{{2f}_{1}}$/${A}_{{f}_{1}}$ is a strong function of t_A_ (relative to $T$). In a conventional oscillator without the additional feedback, ${\tau }_{1}={\tau }_{2}$ which indicates that the oscillator relaxes with a single time constant. However, as ${\tau }_{2}$, and consequently, t_A_ is progressively reduced using the additional diode-connected transistor element in EAO design, the relative amplitude, and thus, the power concentrated at the second harmonic, steadily increases. We also note that the asymmetric output of the EAO (as well as the conventional oscillator used for comparison), characterized by a short rising time (compared to the voltage relaxation time) helps concentrate more power at the second harmonic in comparison to the symmetric waveform (not shown here).

Thus, by engineering t_A_ to be small, a significant portion of the EAO power can be concentrated at the second harmonic. Consequently, the EAO effectively self-generates a strong second harmonic signal in the network, and facilitates the bipartition in the oscillator phases without the need for external second harmonic injection.

**Figure 3:**
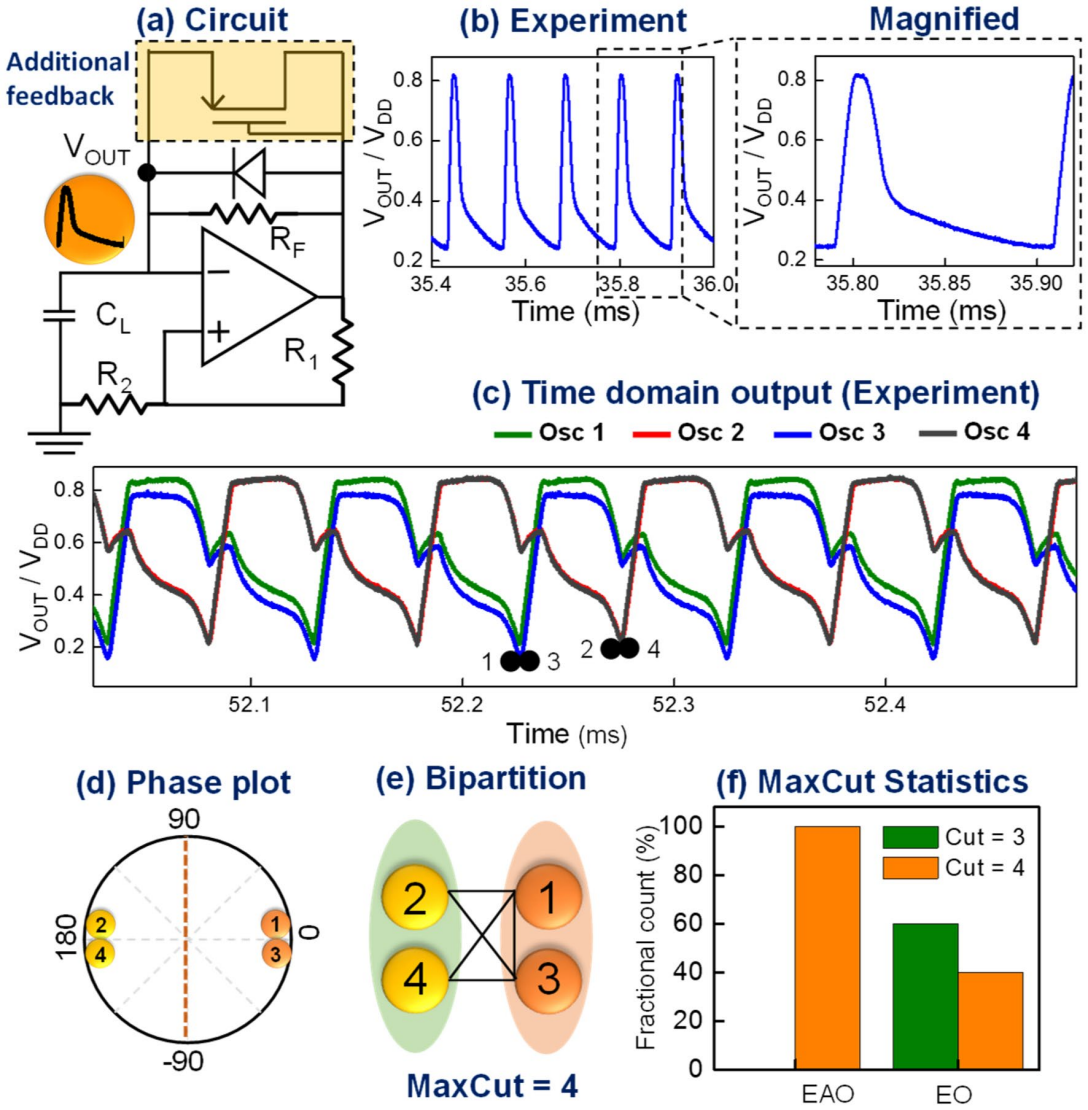
Realizing oscillator based Ising machines without second harmonic injection. (**a**) Schematic; and (**b**) time domain output of the proposed EAO. A magnified image of a single oscillation period revealing the two time constants in the relaxation dynamics is also shown. (**c**) Schematic of the input graph (same as in Fig. 1) and the corresponding experimentally measured time domain output of the equivalent coupled EAO circuit showing the observed phase bipartition. (**d**) Phase plot; and (**e**) Corresponding sets (S1, S2) created by the (Max-) Cut; (**f**) 2D bar plot of MaxCut solutions measured over 10 runs using network of EAOs, and the conventional oscillators (under second harmonic injection).

Next, we experimentally explore the synchronization dynamics of the EAOs by considering the same representative graph as shown in Fig. 1b, and constructing the corresponding equivalent circuit with the coupled EAOs. Figure 3a shows the schematic of EAO design discussed above and Fig. 3b shows experimentally measured time domain waveform of the EAO with distinct time constants. It can be observed from Fig. 3c–e, that the EAO phases exhibit a bipartition (instead of a continuous distribution) without requiring any external injection; in other words, the EAO-based coupled oscillators effectively behave as a system under second harmonic injection, without actually requiring any external injection. Further, we measure the MaxCut solutions obtained with the coupled systems of EAO- and conventional oscillators over multiple trials (Fig. 3f) since Ising machines are known to exhibit statistical behavior owing to the system getting trapped in local minima of the high dimensional phase space. Although for the specific graph instance, we observe that the EAO-based system always computes the optimal solution, we observe similar statistical behavior in larger graph instances considered in the following sections. This performance aspect will be investigated further in future work.

**Figure 4:**
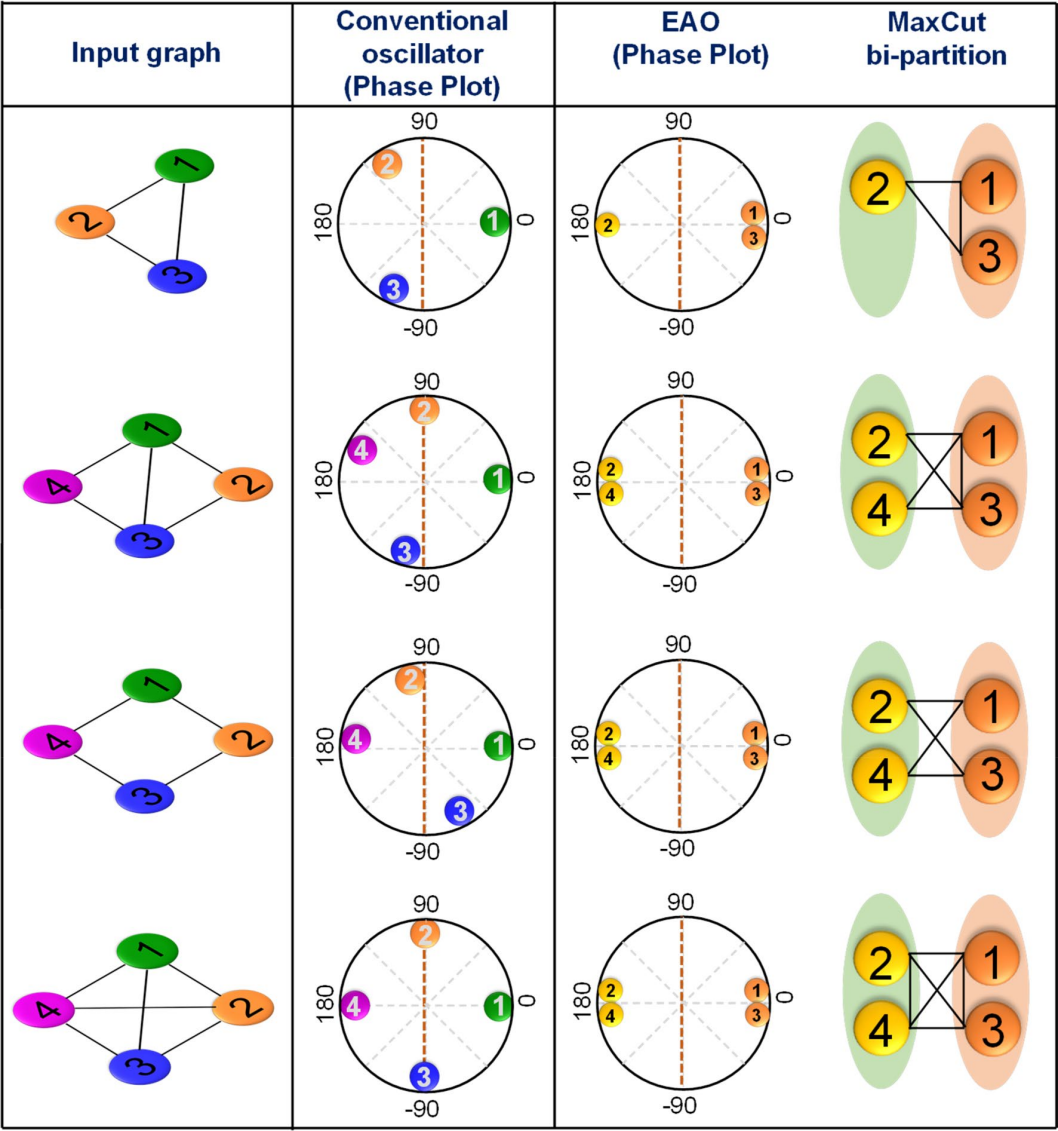
Solving MaxCut using coupled EAOs. Experimentally measured MaxCut solutions for various graph instances obtained using coupled EAOs. The coupled EAOs exhibit a phase bipartition and compute the MaxCut without the need for external second harmonic injection. Corresponding phase dynamics obtained using networks of conventional oscillators (without second harmonic injection) are also shown for comparison. It can be observed that the oscillators do not exhibit a phase bipartition in this case.

Furthermore, we also measure and compare the dynamics of the EAO oscillators with the conventional oscillator design for various graph configurations as shown in Fig. 4. These configurations also exhibit the same bipartition behavior as described earlier as well as show the capability (albeit statistically) to compute the optimal MaxCut solution.

### Solving larger graphs with EAOs

**Figure 5:**
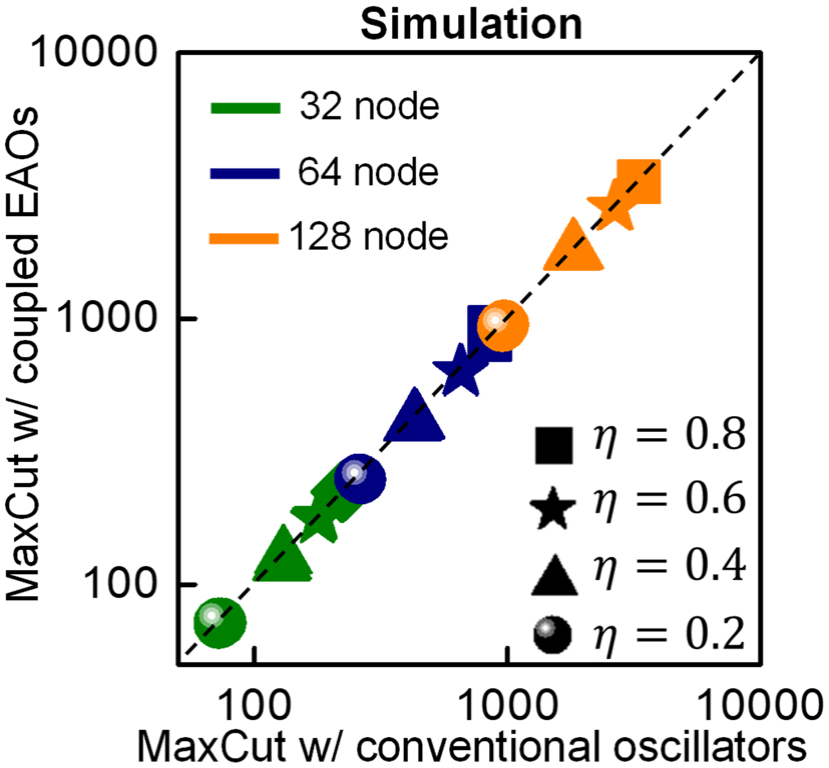
Evaluation of the scalability of autaptic oscillator functionality using simulations. Bubble plot comparing the MaxCut solution obtained with the coupled EAOs (without external injection) with that obtained using coupled networks of conventional oscillators (under external second harmonic injection). Randomly instantiated graphs of various size (V: 32, 64, 128) and edge density (ƞ = 0.2, 0.4, 0.6, 0.8) are considered; 2 graphs are analyzed for every combination of V, ƞ; each graph is simulated 10 times. The results are obtained using simulations performed using SPICE. It can be observed that the MaxCut solutions produced by the oscillators are similar to those obtained using conventional oscillators.

Finally, we evaluate using SPICE-based circuit simulations, the functionality of the autaptic oscillators in larger graphs. We analyze randomly generated graph instances of various size (V: 32, 64, 128) and edge densities (ƞ = 0.2, 0.4, 0.6, 0.8; ƞ: ratio of the number of edges in the graph to the number of edges in a complete graph with the same number of nodes) and compare the results with conventional oscillators operating under second harmonic injection; 2 graphs are analyzed for every combination of V, ƞ; each graph is simulated 10 times. Figure 5 shows a bubble plot comparing the MaxCut solution obtained from the coupled EAOs with that obtained using the conventional oscillators under the influence of second harmonic injection. It can be observed from the simulations that the autaptic oscillators enable the same computational functionality and similar performance as the conventional oscillators under the influence of external second harmonic injection. The EAO based approach exhibits a deviation of − 6.2% to + 9.15% in comparison to the conventional oscillator-based method (+ ^ive^ indicates that the EAO solution is better than that produced by the conventional oscillators), with an average deviation of ~ − 1.6%. This suggests that electronic autaptic oscillator approach can potentially be scaled further although factors such as noise and the coupling architecture will be important to the eventual scalability of the approach. Additionally, we also note that while the proposed EAO helps reduce the cost of implementing the second harmonic signal generation and routing circuitry, other implementation costs such as those associated with the design of the coupling network, which exhibits a square law dependence on the graph size, will also be vital to the feasibility and the competitiveness of the electronic oscillator-based approach to implementing Ising machines as a whole.

## Discussion

The electronic autaptic oscillator concept demonstrated here is a manifestation of a compute-centric device optimization approach to overcome challenges and enable efficient implementation of oscillator-based Ising machines. The feedback methodology used here is not limited to the Schmitt trigger oscillator and can be used to augment other oscillator designs. Considering that area and scalability are key questions in realizing parallel computational architectures like coupled oscillators, this work marks an important step towards improving the scalability and reducing the area requirements for oscillator-based Ising machines.

## Methods

The EAO as well as conventional oscillators for the experimental demonstration were implemented using LM741 OPAMP based Schmitt trigger where oscillations were stabilized using negative feedback. For the conventional oscillator, the negative feedback was implemented using a parallel combination of a feedback resistor (R_F_ = 80 kΩ) and a diode; the diode was used to create an asymmetric waveform with a small rise time, and a relatively large decay to help accentuate the effect of two decay constants in the EAO design. The EAO oscillator also used an additional feedback element implemented using P-MOS (ALD1107) to help generate the two decay time constants. A supply voltage of 8 V was used. The oscillators were assembled on a breadboard and coupled using discrete capacitors in order facilitate the prototypical demonstration. Simulations utilized in the work were performed using SPICE.

## Data Availability

The datasets generated during and/or analyzed during the current study are available from the corresponding author on reasonable request.

## Code availability

All codes used in this work are either publicly available or available from the authors upon reasonable request.
